# Evaluation of dental practitioner habits with occlusal assessment and the clinical application of practical techniques in occlusion, amongst a cohort of participants based in the UK, South Africa, Malta and Malaysia

**DOI:** 10.1111/joor.13358

**Published:** 2022-07-27

**Authors:** Shamir B. Mehta, Daphne Rizzo, Bryan Paulose, Alisa Botbol, Sadhvik Vijay, Amar Arjuna, Subir Banerji

**Affiliations:** ^1^ Department of Conservative & MI Dentistry, Unit of Distance Learning, King's College London Faculty of Dentistry Oral & Craniofacial Sciences London UK

**Keywords:** articulators, centric relation, facebow, occlusal examination, occlusion, undergraduate dental education

## Abstract

**Background:**

Currently, there is a lack of data relating to dental practitioners' habits with clinical occlusal assessment and the application of practical techniques in occlusion.

**Objectives:**

The aim of this study was to investigate habits with clinical occlusal assessment and the practical application of established concepts in occlusion amongst a cohort of international dentists.

**Methods:**

A piloted questionnaire with 20 statements was distributed by 5 recruiters. The recruiters were based in Malta (1), South Africa (1), Malaysia (1) and the UK (2). Outcomes were analysed using descriptives, chi‐squared and Fisher's exact test. All the analyses were carried out in Stata, Version 12. Significance was inferred at *p* < .05.

**Results:**

Four hundred thirty‐five completed responses were included in the sample (response rate, 70.7%). Overall, high levels of agreement were reported with the provision of single‐unit crown and onlay restorations (78.8%) and bridge prostheses (up to 3 units, 77.9%), respectively. One‐third (33.6%) agreed to observing dynamic occlusal relationships during an adult patient dental examination, 40.7% reported using articulators for crown and bridge cases, and 25.1% agreed to taking facebow records. Under half (47.3%) of the dentists expressed dissatisfaction with their undergraduate training in occlusion, with no significant association with the variables of the number of years of experience, the country of practice or being in general practice (*p* ≥ .226).

**Conclusion:**

The results indicate a disparity between traditionally taught and applied concepts in clinical occlusion and the undertaking of occlusal assessments and the management of occlusion in clinical practice.

## Graphical abstract

An evaluation of dental practitioner habits with occlusal assessment and the clinical application of practical techniques in occlusion, amongst a cohort of participants based in the UK, South Africa, Malta and Malaysia.

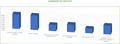

## 1 BACKGROUND

The term ‘occlusion’ has been defined as the static relationship between the incising or masticating surfaces of the maxillary or mandibular teeth, or tooth analogues.^1^ Static occlusion is used to define those tooth contacts between teeth when the mandible is closed and stationary; the term dynamic occlusion is used to describe the contacts between the teeth when there is a movement of the mandible relative to the maxilla.^2^ Dynamic occlusion is influenced by neuromuscular control, by the temporomandibular joints (the posterior determinants) and the occlusal surfaces of the teeth (the anterior determinants).^3^, ^4^ Dental practitioners require a clear understanding and should be able to apply the principles of occlusion to enable them to appropriately restore, reposition and replace teeth.^5^ The importance of developing adequate knowledge and skills with occlusion is underpinned by the explicit requirements set by some of the governing dental councils for undergraduate teaching in this subject area.^5^

There is, however, considerable ambiguity with the topic of dental occlusion. A lack of consensus exists amongst clinicians with the applied concepts and the desired outcomes, especially when undertaking more challenging restorative procedures.^6^ The standard of training and education about occlusal principles, the lack of appropriate scientific evidence to support a plethora of opinion‐based occlusion‐related philosophies with a specific occlusal scheme being superior with the improvement of stomatognathic function^7^ and inconsistency with the associated nomenclature with key terms such as ‘centric occlusion’ and ‘centric relation’^8^ are some factors which have contributed to this overall confusion.

Previous investigations into the teaching of occlusion at undergraduate level^5^, ^9^, ^10^ have identified the need for standardised, clear and contemporaneous teaching guidance. The approach of teaching occlusion by the different disciplines within the profession, sometimes with conflicting ideas and the lack of coordination and consensus between them, may also be a barrier against the effective learning of the accepted concepts in occlusion during undergraduate training.^5^ Variations have also been described in the undergraduate teaching hours devoted to occlusion between dental schools based in the UK and Ireland, as well as in the United States.^5^, ^9^ O'Carroll et al.^5^ also reported differences between the various dental schools with the application of teaching materials and methods, the frequency of taking jaw relationship records and the assessment of competency with occlusion.

Depending upon the type, dental articulators may be used to simulate some or all mandibular movements. This information may be used to facilitate examination of the occlusion and for the fabrication of restorations and prosthesis. There are a variety of dental articulators available in the marketplace.^11^ Whilst guidance is available with the selection of an articulator for a given purpose, currently, there is a lack of scientific evidence to support the selection of an articulator.^12^, ^13^ The use of semi‐adjustable or fully adjustable articulators may provide a more accurate representation of the condylar angle and the relationship between the maxillary plane and the terminal hinge compared with average value articulators or simple hinge articulators. The appropriate use of some articulators may necessitate the taking of a facebow record and/or the taking of a jaw relation.^11^ Although a very high level of consistency has been reported with the teaching of the use of the facebows, the same investigation alluded to the presence of variations in obtaining these records in the clinical setting.^5^ In addition, the frequency of routinely using semi‐adjustable articulators for treatment planning/occlusal assessment and for the execution of care involving single‐unit fixed prosthodontics amongst some of the dental schools based in the UK and Ireland has also been described to be inconsistent.^5^ However, a former investigation into the use of articulators amongst the UK dental schools reported most of them follow the current guidelines and good practice for articulator selection, with the semi‐adjustable type of articulator being the most recommended form of device.^13^

The recording of ‘occlusion’ and ‘occlusal abnormality’ as ‘aspirational’ and ‘conditional’ assessments for all new and recalled dental patient examination appointments, respectively, is advocated by consensus‐based dental record‐keeping standards, NHS England, 2019.^14^ For a new patient attendance (as part of essential practice), the occlusal examination may include an evaluation of the occluding surfaces of the teeth, the incisal angle and molar relationships and the tooth‐related guidance during lateral excursive and protrusive mandibular movements.^15^ However, information relating to the level of undertaking and recording an occlusal examination in clinical practice is limited. This study presents the results following the use of a piloted questionnaire aimed to investigate the habits of a convenience sample of dental practitioners located in one of four countries with the taking of occlusal assessments, the prescription of a variety of treatments that are likely to directly involve a patient's occlusal scheme, the taking of facebow records and the use of semi‐adjustable articulators for fixed prosthodontic (crown and bridge) treatments. As a secondary aim, this study also looked at the participant's satisfaction with their undergraduate training in occlusion.

## 2 METHODS AND MATERIALS

A convenience sample of dental practitioners was recruited by five student volunteers (the ‘recruiters’). The recruiters were based in Malta (DR), Malaysia (BP), South Africa (AB) and the UK (SV and AA). Each recruiter was associated with the MSc in Aesthetic Dentistry programme (AES) at King's College London, Faculty of Dentistry, Oral & Craniofacial Sciences, London, UK. Data collection took place between February 2020 and October 2021. Ethics approval for this investigation was granted by the King's College London, Research and Ethics Committee (MRA‐19/20–17 512). The second layer of recruiters, comprising dental practice managers and dental training course providers, had been identified by the Recruiters. The former recruited a convenient sample of participants. Criteria for inclusion were current registration with a dental council, fluency with spoken English and the ability to complete the questionnaire at the material time. Participants who declined consent were excluded. The participants did not have a clear affiliation with the AES programme of study.

The participant dentists were asked to complete a printed questionnaire, comprising 20 questions. To ensure consistency and accuracy, the questionnaire was piloted amongst a cohort of Year 1 MSc AES students (2019 intake, including 21 dentists). The responses were collected by the second layer of recruiters the same day distribution took place. Responses were subsequently returned to the Recruiter by the electronic scanning of the documents, or in physical format, ensuring the responses remained fully anonymised.

**FIGURE 1:**
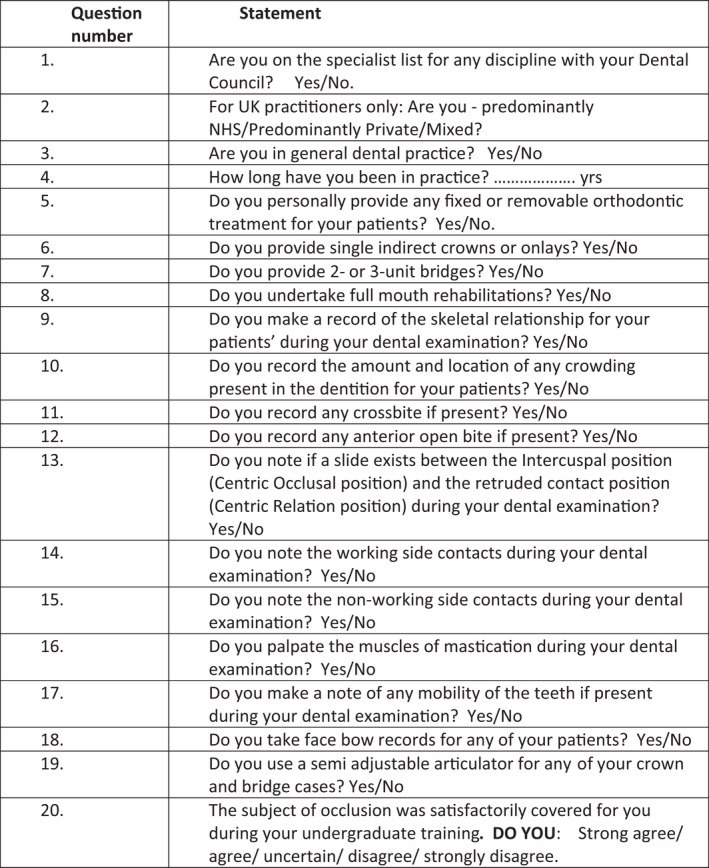
Example of the statements contained in the Questionnaire used in the investigation.

Examples of the statements contained within the questionnaire can be seen in Figure 1. The first four questions (Q1–Q4) related to general demographic aspects, such as the number of years in practice, specialist registration with a dental council (irrespective of the discipline) and presence in general dental practice/the primary dental care setting, and for the UK participants, the arrangements under which they usually provided dental care (state‐funded, private or mixed arrangements). Questions 5 to 8 related to the types of dental treatments provided by the participants for adult patients aged 18 years and over. The latter included the direct provision of fixed or removable orthodontic therapy (Q5) and the undertaking of fixed indirect prosthodontic treatments, thus—single‐unit crown and onlay restorations, 2‐ or 3‐unit fixed dental bridge restorations and full‐mouth rehabilitation (Q6–Q9). A further set of questions (Q9–Q12) related to the recording of aspects of the occlusal assessment during a dental examination for adult dental patients aged 18 years and over, hence, skeletal relationships (Q9) and the recording of some static occlusal relationships—the amount and location of any dental crowding (Q10), the presence of any crossbites (Q11) and the presence of any anterior open bite relationships (Q12). Aspects relating to the observation of dynamic occlusal relationships included noting the presence of a slide between the maximum intercuspal position (centric occlusal position) and the retruded contact position (centric relation position) (Q13) and the presence of any working and non‐working side contacts (Q14 and Q15 respectively). Undertaking the palpation of the muscles of mastication and noting the mobility of teeth (if present) for adult dental patients during a dental examination were addressed by Q16 and Q17 respectively.

The penultimate statements in the questionnaire (Q18 and Q19), respectively, enquired about the taking of facebow records (irrespective of the type of treatment being planned) and the use of semi‐adjustable articulators for any crown and bridge case, again relating to care provided to adult dental patients aged 18 years and over. Question 20 addressed the participants views about the subject of occlusion being satisfactorily covered as part of their dental undergraduate training. Answers to questions 5 to 19 required ‘yes’/or ‘no’ responses; the response to question 20 was based on the Likert's scale, with 5 possible answers.^16^

### 2.1 Statistical analyses

Descriptive statistics such as means, standard deviations, medians and Inter Quartile Ranges were used to summarise the quantitative variables. The categorical variables were summarised using frequencies and cumulative percentages. Cross tabulation was used to determine the association between two categorical variables and other measures, hence the number of years of experience and being in general dental practice. The association between the latter variables with other measures was tested using the chi‐squared test. In the event of any of the cell frequencies being less than 5, Fisher's exact test was applied. Statistical significance was assumed at the 5% (*p* < .05) level. All the analyses were carried out in Stata, Version 12.

## 3 RESULTS

In total, 631 questionnaires were distributed between the five Recruiters (UK—161, South Africa—151, Malta—184 and Malaysia—135); 446 responses were attained, with an overall response rate of 70.7%. Eleven participants were subsequently excluded due to the submission of improperly completed questionnaires, with either one or more missing response and/or where the response(s) provided were illegible. This culminated in an overall study sample of 435 participants.

The participant dentists were based in the UK (108, 24.8%), South Africa (111, 25.6%), Malta (102, 23.4%) and Malaysia (114, 26.2%). The UK sample had been recruited by 2 recruiters (AA and SV), with 47 and 61 participants recruited respectively. Overall, 24 (5.5%) of the participants were registered specialists with a dental council: UK (5, 1.1%), South Africa (16, 3.7%) and Malta (3, 0.7%). There were no registered specialists amongst the Malaysia‐based participants. Overall, 411 (94.5%) of the participants reported being in general dental practice. Amongst the UK‐based sample, 38 (35.2% of the total UK participants) and 27 (25% of the total UK participants) predominantly worked in state‐funded practices (UK National Health Service—NHS) or in privately funded settings, respectively. Of the remaining UK participants, 43 (39.8% of the UK participants) predominantly worked in mixed practice settings.

Overall, the participant's number of years in practice ranged from 1 to 48 years (median, 8 years), with an overall sample mean of 11.4 ± 10.2 years. The mean number of years in practice by country was the UK (13.5 ± 10.2 years), South Africa (11.7 ± 10.25 years), Malta (15.8 ± 11.9 years) and Malaysia (5.4 ± 4.0 years).

Table 1 includes a summary of the participant's responses, where agreement was expressed to a statement within the questionnaire (answer of ‘yes’). Concerning treatments provided for adult dental patients aged 18 years or more, overall 192 (44.1%) of the participants reported directly providing fixed or removable orthodontic treatments, 343 (78.8%) provided single‐unit, indirectly fabricated crown and onlay restorations, 339 (77.9%) provided 2‐ and 3‐unit fixed bridge restorations, and 191 (43.9%) undertook full‐mouth rehabilitation treatments. Differences between the participants with single‐unit crown and onlay treatments were significantly higher for the UK‐based participants (*p* < .0001), with 105 (97.2%) of the UK‐based dentists providing this form of dental care (range between participants from the four countries, 18.8%). Differences between the dentists from each of the four countries with the provision of the other three types of treatment included in the questionnaire were also significant (*p* ≤ .005). The Malta‐based participants reported the highest percentage agreements for providing direct orthodontic treatments (53.9%; range, 16.2%), 2‐ and 3‐unit fixed bridge restorations (96.1%; range, 42.6%) and full‐mouth rehabilitations respectively (63.7%; range, 42.2%).

**TABLE 1:** Summary of the participants agreeing to provide certain types of dental treatments, Q5–Q8. *n* = 435

	South Africa	Malta	UK	Malaysia	Overall	*p*‐value
	*N* (%)	*N* (%)	*N* (%)	*N* (%)	*N* (%)	
Provide any fixed or removable orthodontic treatment for your patients	44 (39.64)	55 (53.92)	50 (46.30)	43 (37.72)	192 (44.14)	<.0001*
Provide single indirect crowns or onlays	87 (78.38)	90 (88.24)	105 (97.22)	61 (53.51)	343 (78.85)	<.0001*
Provide 2‐ or 3‐unit bridges	86 (77.48)	98 (96.08)	94 (87.04)	61 (53.51)	339 (77.93)	<.0001*
Undertake full‐mouth rehabilitations	49 (44.14)	70 (68.63)	42 (38.89)	30 (26.23)	191 (43.91)	.005*

* Indicates differs significantly between countries at .05 level.

For the recording of the skeletal relationships and the recording of static occlusal features during an adult dental patient examination, the overall number of dentists expressing agreement was skeletal relationships—227 (52.2%), the amount and location of crowding in the dentition—293 (67.4%), the presence of a crossbite—353 (81.2%) and the presence of an anterior open bite—372 (85.5%) (Table 2). Significant differences were only seen between dentists from the four countries with the recording of the amount and location of any crowding in the dentition (*p* = .021), and recording the presence of a crossbite (*p* = .008). The South Africa‐based participants showed the highest percentage agreement for the recording of the amount and location of crowding in the dentition (72.1%), the presence of a crossbite (90.1%) and the presence of an anterior open bite (93.7%).

**TABLE 2:** Summary of responses to Q9–Q19, where agreement was expressed (answered ‘yes’) regarding their adult dental patients, *n* = 435

	South Africa	Malta	UK	Malaysia	Overall	*p*‐value
	*N* (%)	*N* (%)	*N* (%)	*N* (%)	*N* (%)	
Record skeletal relationships during dental examination	64 (57.66)	65 (63.73)	50 (46.30)	48 (42.11)	227 (52.18)	.468
Record the amount and location of any crowding present in the dentition	80 (72.07)	70 (68.63)	72 (66.67)	71 (62.28)	293 (67.39)	.021*
Record any crossbite if present	100 (90.09)	83 (81.37)	80 (74.04)	90 (78.95)	353 (81.15)	.008*
Record any anterior open bite if present	104 (93.69)	90 (88.24)	86 (79.63)	92 (80.70)	372 (85.52)	.066
Note if a slide exists between the intercuspal position (Centric Occlusal position) and the retruded contact position (Centric Relation position) during your dental examination?	46 (41.44)	37 (36.27)	27 (25.00)	35 (30.70)	145 (33.33)	.194
Note the working side contacts during your dental examination	41 (36.94)	42 (41.18)	30 (27.78)	45 (39.47)	158 (36.32)	.797
Note the non‐working side contacts during your dental examination	36 (32.43)	38 (37.25)	34 (31.48)	36 (31.58)	144 (33.10)	<.0001*
Palpate the muscles of mastication during your dental examination	74 (67.27)	47 (46.53)	92 (85.19)	39 (3421)	252 (58.20)	.001*
Make a note of any mobility of the teeth if present	109 (98.20)	101 (99.02)	98 (90.74)	113 (99.12)	421 (96.78)	<.0001*
Take facebow records for any of your patients	20 (18.02)	31 (30.39)	45 (41.67)	13 (11.40)	109 (25.06)	<.0001*
Use a semi‐adjustable articulator for any of your crown and bridge cases	42 (37.84)	56 (54.90)	51 (47.22)	28 (24.56)	177 (40.69)	<.0001*

* Indicates differs significantly between countries at .05 level.

For the dynamic occlusal features observed during an adult patient dental examination, overall, 145 (33.3%) of the dentists agreed observing for the presence of a slide between the centric occlusal position and the centric relation position, whilst 158 (36.3%) and 144 (31.3%) of them respectively noted the presence of working and non‐working side contacts. As illustrated in Table 2, significant differences between the participants from the various countries were only seen for observing the presence of non‐working side contacts (*p* < .0001).

Concerning the palpation of the muscles of mastication and noting the mobility of any teeth (if present) during an adult patient dental examination, overall, 252 (58.2%) and 421 (96.8%) of the dentists respectively expressed agreement. Significant differences between participants from each country were also observed for these two variables (*p* = .001 and *p* < .0001) respectively.

In total, 109 dentists (25.1%) took facebow records and 177 (40.7%) of them reported using semi‐adjustable articulators for any crown and bridge case. Significant differences between participants from the four different countries represented in the study sample were also noted (*p* < .001, for each of these two variables). Levels of agreement with the taking of facebow records ranged from 41.7% amongst the UK‐based participants to 18.0% for the South Africa‐based participants, and for the use of a semi‐adjustable articulator for a crown and bridge case, from 54.9% (Malta‐based participants) to 37.8% (South Africa‐based participants).

Table 3 provides a summary of the levels of satisfaction expressed with the subject of occlusion during undergraduate training. For the overall sample, 38 (8.8%) of the dentists ‘strongly agreed’ and 115 (26.4%) ‘agreed’ with the statement in Q20, thus collective ‘overall agreement’ expression by 153 (35.1%) of the participants. ‘Uncertainty’ with this statement was recorded by 76 (17.5%) dentists. ‘Disagreement’ with this statement was expressed by 155 (35.6%) dentists, and 51 (11.7%) of them ‘strongly disagreed’, culminating in an expression of ‘overall disagreement’ by 206 (47.3%) dentists. The majority of the participants from Malta and Malaysia (60.8% and 74.9% respectively) collectively expressed uncertainty or some level of disagreement with this statement. In contrast, a higher percentage of UK‐based participants (57, 52.7%) reported the subject of occlusion was satisfactorily covered in their undergraduate training.

**TABLE 3:** Subject of occlusion was satisfactorily covered during undergraduate training, Q20

	South Africa	Malta	UK	Malaysia	Overall
	*N* (%)	*N* (%)	*N* (%)	*N* (%)	*N* (%)
Strongly agree	10 (9.01)	1 (0.98)	19 (17.59)	8 (7.02)	38 (8.74)
Agree	31 (27.93)	20 (19.61)	38 (35.19)	26 (22.81)	115 (26.44)
Uncertain	11 (9.91)	25 (24.51)	11 (10.19)	29 (25.44)	76 (17.47)
Disagree	44 (39.64)	37 (36.27)	29 (26.85)	45 (39.47)	155 (35.63)
Strongly disagree	15 (13.51)	19 (18.63)	11 (10.19)	6 (5.26)	51 (11.72)

Depicted by Table 4 is the association between the number of years in practice with other factors. A significant and positive effect was observed between the number of years in practice and the variables of specialist registration (*p* = .005), as well as the types of treatment provided—either single‐unit indirect crowns or onlays, 2‐ or 3‐unit fixed bridge restorations and the undertaking of full‐mouth rehabilitations (*p* < .001). Significant associations between the number of years in practice and the recording of skeletal relationships (*p* < .001), recording the presence of a crossbite (*p* = .013), the taking of facebow records (*p* < .0001) and the use of a semi‐adjustable articulator for any crown and bridge case (*p* = .001) were also reported. For each of the latter findings, an increase in the number of years in practice culminated in a higher outcome.

**TABLE 4:** Association between number of years in practice with other variables

Measures	*p*‐value
On the specialist list for any discipline with your Dental Council	.005*
Provide single indirect crowns or onlays	<.0001*
Provide 2‐ or 3‐unit bridges	<.0001*
Undertake full‐mouth rehabilitations	<.0001*
Providing fixed or removable orthodontic treatment to the patients	.733
Make a record of the skeletal relationship for your patients' during your dental examination	<.0001*
Record the amount and location of any crowding present in the dentition for your patients	.276
Record any crossbite if present	.013*
Record any anterior open bite if present	.148
Note if a slide exists between the intercuspal position (centric occlusal position) and the retruded contact position (centric relation position) during your dental examination	.088
Note the working side contacts during your dental examination	.779
Note the non‐working side contacts during your dental examination	.436
Palpate the muscles of mastication during your dental examination	.079
Note if any mobility of the teeth if present during your dental examination	.76
Take facebow records for any of your patients	<.0001*
Use a semi‐adjustable articulator for any of your crown and bridge cases	.001*
Subject of occlusion was satisfactorily covered for you during your undergraduate training	.923

* Denotes statistical significance.

Table 5 shows the association between being in general dental practice with various factors. Amongst the sixteen potential factors considered, significant associations with this variable included specialist registration (*p* < .001), the provision of either single‐unit crown and onlay restorations and 2‐ and 3‐unit fixed bridge restorations (*p* ≤ .002), recording of skeletal relationships (*p* = .021), observing the presence of a slide between the centric occlusal position and the centric relation position and noting the presence of working and non‐working side contacts during an adult patient dental examination (*p* ≤ .008). For each of the latter significant associations, the effect of being specialist registration resulted in a higher outcome.

**TABLE 5:** Association between general dental practice with other measures

Measures	*p*‐value
On the specialist list for any discipline with your Dental Council	<.0001*
Provide single indirect crowns or onlays	.002*
Provide 2‐ or 3‐unit bridges	.017*
Undertake full‐mouth rehabilitations	.845
Providing fixed or removable orthodontic treatment to the patients	.273
Make a record of the skeletal relationship for your patients' during your dental examination	.021*
Record the amount and location of any crowding present in the dentition for your patients	.578
Record any crossbite if present	.28
Record any anterior open bite if present	.228
Note if a slide exists between the intercuspal position (Centric Occlusal position) and the retruded contact position (Centric Relation position) during your dental examination	.008*
Note the working side contacts during your dental examination	.006*
Note the non‐working side contacts during your dental examination	.002*
Palpate the muscles of mastication during your dental examination	.086
Make a note of any mobility of the teeth if present during your dental examination	.554
Take facebow records for any of your patients	.148
Use a semi‐adjustable articulator for any of your crown and bridge cases	.598
Subject of occlusion was satisfactorily covered for you during your undergraduate training	.226

* Denotes statistical significance.

## 4 DISCUSSION

A convenience sample of dentists from four countries reported relatively high levels of agreement with the provision of single‐unit crown and onlay restorations (78.8%) and bridge prostheses (up to 3 units, 77.9%) respectively. However, only one‐third (33.6%) agreed observing dynamic occlusal relationships during an adult patient dental examination, 40.7% reported using articulators for crown and bridge cases, and 25.1% agreed with the taking of facebow records. High levels of agreement were also observed for the recording of static occlusal features during an adult dental patient examination appointment (the location and amount of any dental arch crowding, a crossbite and/or an anterior open bite; mean for these three factors—78.0%). Overall, just under half (47.3%) of the dentists expressed dissatisfaction with their undergraduate training in occlusion, with no significant association with the variables of the number of years of experience, the country of practice or being in general practice (*p* ≥ .226). The results of this study support the need for improvement in the understanding and application of traditional occlusal concepts in clinical practice.

A questionnaire may be used to estimate practising trends and professional perceptions. However, it is not free of inaccuracy, with the risk of attaining socially desirable responses. The current questionnaire was largely limited to closed responses and did not include a comprehensive overview of the concepts and practice of clinical occlusion. Statements included were also limited to care provided for adult dental patients during a routine dental examination, and some of them related to record keeping. Some clinicians may perform assessments and evaluations that are not routinely recorded in their contemporaneous patient notes. The convenience sample in this study, drawn from a heterogeneous group of dental practitioners, also largely consisted of relatively younger dental practitioners, with a mean 11.4 ± 10.2 years of experience and a significant representation of participants in general dental practice (*p* = .007). The participants in the present study can neither be considered fully representative of the dental profession for each country; however, the data gathered in this investigation may give some indication of the views and attitudes of dentists about the subject of clinical occlusion.

The provision of fixed prosthodontic treatment has formerly been reported to be undertaken by 67% of general dental practitioners in South East England^17^; these outcomes are comparable to the overall sample data from this investigation. Although the restoration of a limited number of occluding surfaces may appear simple, often requiring a conformative approach, in order to help ensure the unwanted alteration of the static and dynamic occlusal relationships pre‐ and post‐treatment,^18^ there would be the need to undertake an appropriate appraisal of the dynamic occlusal relationships.^2^ The latter would include attempting to identify working and non‐working side occlusal contacts. An overview of the factors that may influence the prescription of a confirmative approach, or when this may not be appropriate, the decision with how and when to re‐organise the occlusion has been documented in the contemporary literature.^18^ In the present study, routine fixed indirect prosthodontic treatments were provided by over three‐quarters of the sample; however, only one‐third agreed undertaking dynamic occlusal assessments. The precise reasons for a disparity in the numbers of dentists providing single‐unit crown and onlay restorations and those undertaking dynamic occlusal assessments are unknown. Accepting the limitations of this study, this divide may be indicative of the participants' knowledge and practical application of the established concepts in clinical occlusion,^2^ the presence of a possible ‘disconnect’ between the undergraduate prosthodontic curriculum and the general practice of dentistry or the perception of being able to deliver effective and efficient dental care using protocols that are more commonly applied and prescribed in general dental practice.^19^ Disparity between the teaching and application of techniques in dental schools and clinical practice in removable prosthodontics have also been described in the literature.^20^, ^21^, ^22^

Taking of a facebow record and the use of a semi‐adjustable articulator may be indicated with the planning and preparation of fixed (and removable) prosthodontic treatments.^11^ This may include the provision of a single‐unit crown or onlay restorations. All the dental schools in the UK and Ireland that took part in the study by O'Carroll et al.^5^ documented the teaching of the use of a facebow and recording jaw relations, and the use of articulators amongst the UK dental schools has been reported in a different study to closely follow the available guidelines and recommendations.^13^ However, a previous investigation showed that only a small percentage of dentists continued to use or prescribed the use of dental articulators after commencing practice.^23^ Clark et al.^19^ reported 50.6% of a sample of 1265 general dental practitioners based in the United States utilising semi‐adjustable articulators for all removable prosthodontics and extensive crown and bridgework, comprising 5 units or more, and 29.6% agreed to taking of facebow records when fabricating fixed and removable prosthodontic restorations. In the present study, whilst some significant differences were observed amongst the participants from the four countries (*p* < .001), overall, 40.7% of the participants agreed to using a semi‐adjustable articulator for crown and bridge work, with only 25.1% reporting to taking facebow records. The reason for the relatively lower proportion of participants using semi‐adjustable articulators for this application is unknown. However, the current absence of clinical data to support the superiority with the clinical outcome or oral function with the use of an adjustable articulator versus the use of a simpler articulator^7^ may be a key consideration.

The taking of a facebow record would traditionally be indicated for the mounting of a maxillary cast relative to the terminal hinge axis when using a dental articulator. The data from the current study allude to dentists using a semi‐adjustable articulator, without the attainment of a facebow record. This may possibly reflect the participants' knowledge and understanding of occlusion, or perhaps the failure of clinical studies to confirm the superiority of techniques with an improvement in oral function or clinical outcomes involving the use of facebow transfer versus those that do not require it.^7^ It may also be postulated that some of the participants reporting the use of a semi‐adjustable articulator may be delegating the responsibility of selecting and using a dental articulator, entirely to their dental technician. A former study into the analysis of the use of dental articulators and dental education and practice reported 10% of dental practitioners did not state the type of articulator they used to mount their casts on.^24^

The risks of introducing errors with the patient's occlusal scheme may be assumed to be heightened when undertaking more advanced procedures, such as full‐mouth rehabilitation, often involving considerable functional and aesthetic changes. In this investigation, 43.9% of the overall dentists reported providing full‐mouth rehabilitation treatments. With approximately one‐third of the participants observing dynamic occlusal relationships at the time of undertaking examination and only a quarter attaining facebow records, this may be considered a potential area of significant concern.

With general dental practitioners increasingly providing orthodontic services,^23^ it was by no means surprising to see a relatively high proportion of the current study sample (with only 5.5% registered specialists in all disciplines) providing fixed and removable orthodontic treatments (44.1%). Orthodontic intervention would also require appropriate occlusal assessment, with some recording of the static occlusal features.^25^, ^26^ The latter, together with some of the available guidance for clinical examination and record keeping, stipulating the need to appraise the nature of the occlusion as baseline information^27^ may have accounted for the higher number of participants agreeing to the recording of some static occlusal features. Such features are perhaps less burdensome and less time‐consuming to appraise, than for instance, observing the presence of a slide from the centric occlusal position to the centric relation position. Recording of a skeletal assessment during an adult patient dental examination was relatively lower than the proportion of participants recording some static occlusal assessments (52.2% and 78.0% respectively); this difference may be due to skeletal assessment primarily forming part of the clinical orthodontic assessment.^25^

The proportion of dentists agreeing to palpate the muscles of mastication during an adult dental patient (58.2%) was comparable to the outcomes of a former investigation performed amongst a group of Swedish general dental practitioners and dental hygienists.^28^ Assessment of the temporomandibular joints at rest and during mandibular movement is advised by the available UK‐based guidance when performing a routine extra‐oral examination for findings such, as clicking, grating, limitation of movement, effusions, pain or tenderness; however, this guidance does not explicitly include the need for palpation of the muscle of mastication, which may be conditional on presentation.^27^ As tooth mobility may be the manifestation of a plethora of dental conditions (including occlusal disharmony), despite significant differences between dentists from the various countries represented in the current study sample, it was unsurprising to see high levels of agreement (96.8%) with this statement.

The participants in this study with a greater number of years in practice were also significantly more likely to provide more advanced prosthodontic rehabilitation, attain facebow records and use semi‐adjustable articulators (*p* ≤ .001). Contrastingly, a previous investigation reported more recent graduates (1–5 years in practice) to be significantly more likely to use a semi‐adjustable articulator than older graduates (15 years or more in practice).^19^ Based on the outcomes of the present study, it would be plausible to assume more experienced dentists having possibly acquired the necessary, knowledge, skills, competence and training, feeling more confident whilst embarking upon more technically challenging clinical cases, with treatment involving planned changes to a patient's occlusal scheme. As a limitation of this study, the data were not corrected for the variables of age and the complexity of the treatment undertaken.

Some significant differences were also observed between the participants from the four countries. Malta‐based dentists were significantly more likely to provide orthodontic treatment and more advanced prosthodontic care (*p* ≤ .005). The Malta‐based participants in this investigation also had the highest mean experience of years (15.8 ± 11.9 years, versus 5.4 ± 4.0 years for the Malaysia‐based dentists). This investigation reported a significant relationship between the variable of the number of years of experience and the undertaking of more complex types of dental care. The number of dentists per inhabitant in Malta and Gozo has also been identified to be relatively low (1: 1800) compared with other countries in the European Union.^29^ Such dentist to patient ratio may place a greater demand on clinicians to perform more extensive types of dental care and to acquire the necessary skills and competence to enable effective execution of the planned treatment.

Dissatisfaction with the teaching of occlusion at undergraduate level in this study was relatively high (47.3%), with no significant relationship between the number of years of experience or being in general dental practice. Former investigations have also reported poor perceptions of undergraduate education with occlusion, with a lack of adequate training in preparation for clinical practice.^30^, ^31^ The latter findings together with the outcomes of the present investigation support the changes described by O'Carroll et al.^5^ to include an improvement in the consistency with the teaching of this subject and enhanced coordination between the different disciplines involved with the teaching of occlusion.

The outcomes of this investigation have alluded to some important issues with the application of concepts in clinical occlusion. However, given the limitations discussed above, there is a clear need for further research in this field, especially in relation to the evidence‐base to support occlusal practice.

## 5 CONCLUSION

The outcomes of this study allude to a disparity between traditionally applied concepts with the undertaking of occlusal examination and the management of occlusion in clinical practice. The findings from this study support the need for some improvement in these areas in clinical dentistry, with a more effective translation of the knowledge and skills likely to have been acquired during undergraduate training into routine clinical practice.

## AUTHOR CONTRIBUTIONS

**Shamir B. Mehta** contributed to the investigation, writing—original draft preparation, formal analysis, software, supervision, conceptualisation and project administration. **Daphe Rizzo, Alisa Botbol, Bryan Paulose, Sadhvik Vijay and Amar Arjuna contributed to** the methodology, investigation and writing—review and editing. **Subir Banerji contributed to the** supervision, writing—review, project administration and editing, formal analysis, software, conceptualisation and project administration.

## CONFLICT OF INTEREST

The authors did not receive any financial support and declare no potential conflicts of interest with respect to the authorship and/or publication of this article.

### PEER REVIEW

The peer review history for this article is available at https://publons.com/publon/10.1111/joor.13358.

## Data Availability

The data that support the findings of this study are available from the corresponding author upon reasonable request.
